# Genome-Wide Copy Number Analysis Uncovers a New HSCR Gene: *NRG3*

**DOI:** 10.1371/journal.pgen.1002687

**Published:** 2012-05-10

**Authors:** Clara Sze-Man Tang, Guo Cheng, Man-Ting So, Benjamin Hon-Kei Yip, Xiao-Ping Miao, Emily Hoi-Man Wong, Elly Sau-Wai Ngan, Vincent Chi-Hang Lui, You-Qiang Song, Danny Chan, Kenneth Cheung, Zhen-Wei Yuan, Liu Lei, Patrick Ho-Yu Chung, Xue-Lai Liu, Kenneth Kak-Yuen Wong, Christian R. Marshall, Steve Scherer, Stacey S. Cherny, Pak-Chung Sham, Paul Kwong-Hang Tam, Maria-Mercè Garcia-Barceló

**Affiliations:** 1 Department of Surgery, Li Ka Shing Faculty of Medicine, The University of Hong Kong, Hong Kong, China; 2 Department of Psychiatry, Li Ka Shing Faculty of Medicine, The University of Hong Kong, Hong Kong, China; 3 Department of Epidemiology and Biostatistics, School of Public Health, Tongji Medical College, Huazhong University of Science and Technology, Wuhan, China; 4 Centre for Reproduction, Development, and Growth, Li Ka Shing Faculty of Medicine, The University of Hong Kong, Hong Kong, China; 5 Department of Biochemistry, Li Ka Shing Faculty of Medicine, The University of Hong Kong, Hong Kong, China; 6 Department of Orthopedics and Traumatology, Li Ka Shing Faculty of Medicine, The University of Hong Kong, Hong Kong, China; 7 Department of Paediatric Surgery, Shengjing Hospital, China Medical University, Shenyang, China; 8 Department of Surgery, Shenzhen Children's Hospital, Shenzhen, China; 9 Program in Genetics and Genome Biology and The Centre for Applied Genomics, The Hospital for Sick Children, Toronto, Ontario, Canada; 10 The McLaughlin Centre and the Department of Molecular Genetics, University of Toronto, Toronto, Ontario, Canada; 11 Genome Research Centre, Li Ka Shing Faculty of Medicine, The University of Hong Kong, Hong Kong, China; 12 State Key Laboratory of Brain and Cognitive Sciences, Li Ka Shing Faculty of Medicine, The University of Hong Kong, Hong Kong, China; Harvard Medical School, United States of America

## Abstract

Hirschsprung disease (HSCR) is a congenital disorder characterized by aganglionosis of the distal intestine. To assess the contribution of copy number variants (CNVs) to HSCR, we analysed the data generated from our previous genome-wide association study on HSCR patients, whereby we identified *NRG1* as a new HSCR susceptibility locus. Analysis of 129 Chinese patients and 331 ethnically matched controls showed that HSCR patients have a greater burden of rare CNVs (*p* = 1.50×10^−5^), particularly for those encompassing genes (*p* = 5.00×10^−6^). Our study identified 246 rare-genic CNVs exclusive to patients. Among those, we detected a *NRG3* deletion (*p* = 1.64×10^−3^). Subsequent follow-up (96 additional patients and 220 controls) on *NRG3* revealed 9 deletions (combined *p* = 3.36×10^−5^) and 2 *de novo* duplications among patients and two deletions among controls. Importantly, *NRG3* is a paralog of *NRG1*. Stratification of patients by presence/absence of HSCR–associated syndromes showed that while syndromic–HSCR patients carried significantly longer CNVs than the non-syndromic or controls (*p* = 1.50×10^−5^), non-syndromic patients were enriched in CNV number when compared to controls (*p* = 4.00×10^−6^) or the syndromic counterpart. Our results suggest a role for *NRG3* in HSCR etiology and provide insights into the relative contribution of structural variants in both syndromic and non-syndromic HSCR. This would be the first genome-wide catalog of copy number variants identified in HSCR.

## Author Summary

Copy number variations (CNVs) are significant genetic risk factors in disease pathogenesis and represent an important portion of missing heritability for some human diseases, making their discovery essential for the identification of genes and risk factors for a wide range of diseases, including Hirschsprung disease (HSCR, congenital colon aganglionosis). Since the discovery of the major HSCR gene, *RET*, a number of rare mutations have been reported in *RET* and other genes involved in the development of the enteric nervous system. However, these mutations contribute to only a small proportion of the disease susceptibility. Taking advantage of the recent technical and methodological advances, we have examined the contribution of CNVs to the disease. We have found that HSCR patients are enriched with CNVs encompassing genes. In particular, we found that deletions of *NRG3*, a paralog of the previously identified HSCR–susceptibility gene *NRG1*, were associated with the HSCR phenotype.

## Introduction

Hirschsprung disease (HSCR, aganglionic megacolon) is a rare, congenital disorder characterized by the absence of enteric ganglia along a variable length of the intestine. It can be classified according to the length of aganglionosis into short segment (S-HSCR; 80% of the cases), long-segment (L-HSCR; 15%) and total colonic aganglionosis (TCA; 5%). The incidence of Hirschsprung disease varies by gender and ethnicity, and is highest among Asians (2.8/10,000 newborns) [1]. The male∶female ratio is ≈4∶1 among S-HSCR patients and ≈1∶1 among L-HSCR patients. The majority of HSCR patients are isolated (non-syndromic and sporadic) S-HSCR whose modes of inheritance are primarily multifactorial.

Since the discovery of the major HSCR gene, receptor tyrosine kinase (*RET*; 10q11), a number of rare mutations have been reported in genes (*EDNRB*; 13q22, *GDNF*; 5p13, *PHOX2B*; 4p13, *SOX10*; 22q13, etc) mostly involved in the two interrelated pathways: RET and endothelin receptor B (EDNRB) signaling cascade [1]. However, together these mutations are of incomplete penetrance and account for only 50% of the familial (mostly L-HSCR, TCA) and up to 20% of the sporadic cases (mostly S-HSCR), contributing to only a small proportion of the heritability [2]. On the other hand, common variants in *RET* and *NRG1* (8p12) were found associated with all sub-phenotypes and explained a considerably larger variance [3], [4]. Still, in spite of the vast coding sequence (CDS) mutation screening and the genome-wide association mapping on HSCR patients, a substantial genetic contribution remained elusive.

Copy number variations (CNVs), which represent an important portion of missing heritability, have recently been highlighted as significant genetic risk factors in disease pathogenesis, such as schizophrenia, autism and early-onset obesity [5]–[7]. Through these genome-wide CNV analyses, a number of disease-susceptibility genes have been suggested (e.g. *SH2B1* in obesity and *NRXN1* in autism and schizophrenia). In fact, CNV discovery has been essential for uncovering genes/risk factors for a wide range of diseases, including Hirschsprung disease. The two major HSCR genes—*RET* and *EDNRB*—are indeed the classical examples of how structural variations assist in mapping the disease-predisposing genes. It is estimated that about 12% of Hirschsprung patients have structural abnormalities [1]. Among these, trisomy 21 (Down's syndrome) is the commonest anomalies, involving 2–10% of the patients [8]. Given this early impact of CNVs on gene discovery and the non-random association of HSCR with syndromes, it is highly probable that structural variations underlain HSCR. Thus far, several studies have attempted to survey CNVs in the targeted HSCR genes (*RET*, *GDNF*, etc) [9]–[11], albeit the extent to which CNVs contribute to HSCR is still largely unknown.

To systematically explore the global contribution of CNVs to the disease, we performed a genome-wide copy number analysis based on our previously published SNP genotyping data [3]. By performing a comprehensive association analysis on the identified structural variants, we aimed to uncover novel genes conferring risk to HSCR.

## Results

After extensive pre- and post- calling quality control (QC), we obtained a stringent dataset of 866 CNVs with a median size of 34.39 kb in 129 HSCR cases (excluding chromosome 21 for 8 Down's syndrome patients) and 1515 CNVs with a median size of 57.90 kb in 331 ethnically matched controls. Apart from the 1.5 fold increase in average CNV count for cases, a higher proportion of deletions, presumably of larger functional impact, were also observed in cases (59.36%) over controls (51.68%), which allowed us to hypothesize that CNVs significantly contribute to the pathogenesis of HSCR.

To delineate the global impact of CNVs on disease susceptibility, we compared the overall CNV burden in cases relative to controls, in terms of the estimated CNV size, number of CNVs per individual (rate of CNV) and number of genes overlapped by CNVs (gene count).

### Greater burden of rare CNVs in HSCR patients

Rare CNVs (present in <1% of the general population) were found significantly overrepresented in HSCR cases with a ratio of 1.97 (*p* = 1.50×10^−5^; conditional permutation *p* = 4.97×10^−3^). Such difference was not observed for common CNVs, in accordance with their weak global contribution to diseases [12]. As shown in Table 1, the rate of both rare deletions and duplications were significantly higher in HSCR patients; furthermore, these CNVs intersected with more genes. The association was stronger for deletions with a 2.31 fold increase in rate (*p* = 9.20×10^−5^; conditional permutation *p* = 0.017) and with 7.61 times more genes overlapped when compared to controls (*p* = 5.00×10^−6^; conditional permutation *p* = 2.60×10^−5^). In particular, long deletions (>100 kb) were 14 times enriched with genes in cases when compared to controls (*p* = 4.25×10^−4^; conditional permutation *p* = 2.61×10^−4^). This could be partly explained by the increase in number of CNVs per patient (rate) (*p* = 0.014; conditional permutation *p* = 0.051) as well as by the larger size of CNVs (*p* = 0.047; conditional permutation *p* = 0.015) when compared to controls. Consistently, singleton (single occurrence) genic deletions were found more abundant in patients.

**Table 1:** Global CNV burden in HSCR patients.

	Size (kb)				CNV burden (Rate)^a^				CNV burden (Gene count)			
	Baseline^b^	Ratio^c^	*P*	Conditional *P* d	Baseline	Ratio	*P*	Conditional *P*	Baseline	Ratio	*P*	Conditional *P*
	**All CNVs**											
***Frequency***												
Common	141.3	0.72	0.025*	0.98	2.33	0.96	0.61	0.59	2.74	0.90		0.62
Rare	135.0	2.89	4.61×10^−3^ **	1.03×10^−3^ **	2.27	1.97	1.50×10^−5^ ***	4.97×10^−3^ **	1.80	4.13	6.00×10^−6^ ***	1.30×10^−5^ ***
	**Rare CNVs**											
***Size***												
*All* e												
Del	82.2	4.57	0.021*	6.27×10^−3^ **	1.22	2.31	9.20×10^−5^ ***	0.017*	0.54	7.61	5.00×10^−6^ ***	2.60×10^−5^ ***
Dup	179.9	1.51	0.086	0.030*	1.05	1.57	0.019*	0.074	1.26	2.65	8.53×10^−3^ **	0.015*
*Long (>100 kb)*												
Del+Dup	338.6	3.05	0.013*	2.51×10^−3^ *	0.69	1.44	6.77×10^−3^ **	0.020*	1.12	5.01	1.11×10^−4^ **	7.30×10^−5^ ***
Del	286.5	6.54	0.047*	0.015*	0.22	1.78	0.014*	0.051	0.21	14.23	4.25×10^−4^ **	2.61×10^−4^ **
Dup	351.3	1.39	0.12	0.044	0.47	1.27	0.16	0.11	0.92	2.95	0.021*	0.023*
	**Rare CNVs**											
***Occurence***												
*Singleton*												
Del+Dup	148.2	3.53	3.71×10^−3^ **	8.78×10^−4^ **	1.34	2.33	4.30×10^−5^ ***	7.38×10^−3^ **	1.26	5.35	1.10×10^−5^ ***	4.10×10^−5^ ***
Del	84.9	7.19	0.019*	4.75×10^−3^ **	0.76	2.51	5.73×10^−4^ **	0.042*	0.38	9.88	3.90×10^−5^ ***	8.90×10^−5^ ***
Dup	220.2	1.58	0.081	0.041	0.57	2.11	5.22×10^−3^ **	0.029*	0.88	3.42	6.35×10^−3^ **	0.014*
*2–4 times*												
Del+Dup	114.9	5.28	0.025*	9.97×10^−3^ **	0.97	1.42	2.91×10^−3^ **	0.052	0.60	4.24	5.94×10^−3^ **	1.66×10^−3^ **
Del	79.3	7.95	0.14	0.050	0.47	1.97	2.90×10^−4^ **	0.017*	0.17	9.75	8.30×10^−4^ **	9.34×10^−4^ **
Dup	143.6	2.23	0.18	0.14	0.50	0.92	0.72	0.68	0.44	2.14	0.23	0.17

a Rate: number of CNVs per individual.

b Baseline: size, rate or gene count of controls.

c Ratio: Case/control ratio.

d *P*-value for conditional permutation based on score of LRR_SD, BAF_drift and median absolute deviation.

e Del+Dup of all rare CNVs was specified as above.

*: 0.01<*p*<0.05;

**: 10^−4^<*p*<0.01;

***: *p*<10^−4^.

Recognizing that CNV analysis is more sensitive to outliers and batch effects, we evaluated if any of these potential factors might account for the observed CNV burden (Text S1, Table S3, Figures S2, S3 and S4). For both CNV rate and gene count, the distinctive overall CNV distribution in HSCR cases (Figure S6) together with the insignificant association with *Affymetrix* plates confirmed that our findings were not attributed to experimental artifacts. Moreover, the similar level of significance for overrepresentation achieved by conditional permutation on data quality, as illustrated by conditional *p*-value in Table 1, further demonstrated the robustness of our findings.

Summarizing, HSCR patients have more rare-CNVs and more genes intersected by rare CNVs when compared to controls. The overrepresentation of rare genic CNVs in HSCR patients implies that some CNVs could be pathogenic, regardless of the size and occurrence.

### Rare CNVs distribution in syndromic HSCR and isolated (non-syndromic) HSCR patients

We have previously demonstrated that the genetic susceptibility to HSCR varies across sub-phenotypes such as familiality and segment length [2]. To assess if such genetic heterogeneity also occurs at the structural variation level, we further examined the CNV burden for 29 syndromic and 100 non-syndromic (isolated) HSCR separately.

**Figure 1:**
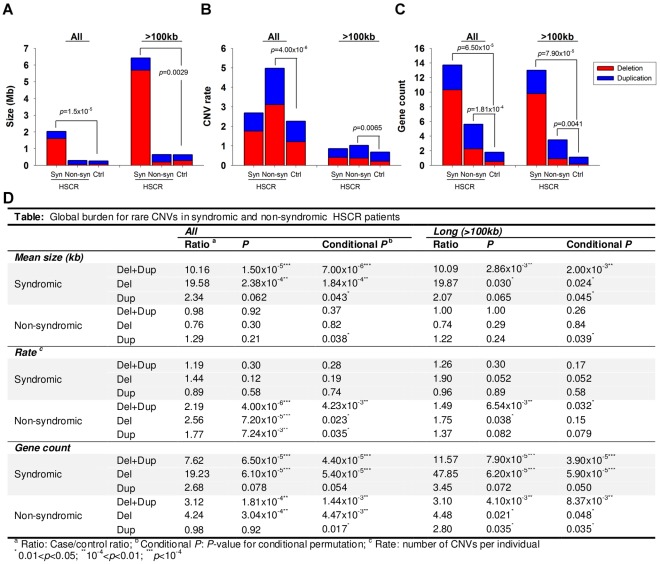
CNV burden for syndromic (Syn) and non-syndromic (Non-syn) HSCR patients relative to controls (Ctrl). The burden was measured with reference to the (A) size, (B) rate and (C) gene count of CNVs. Red and blue bars denote the mean value of the corresponding test for deletion and duplication respectively. Summary statistics as well as conditional permutation *p*-value was shown in (D).

As illustrated in Figure 1, syndromic HSCR cases, on average, harbored longer CNVs than non-syndromic HSCR cases or controls (*p* = 1.50×10^−5^ *vs.* controls) even when the 8 HSCR patients with Down syndrome were excluded from the analysis. Had these patients been taken into account, the significance of the association would have been much lower (*p*<1×10^−8^). On the other hand, non-syndromic HSCR cases were enriched with rare CNVs compared to syndromic patients or controls (*p* = 4.00×10^−6^ *vs.* controls). Whilst the involvement of CNVs is somewhat expected in syndromic patients, the excess of rare CNVs in isolated HSCR, irrespective of the size, suggested that copy number variants also contribute to the manifestation of isolated Hirschsprung disease.

It is tempting to speculate that the CNVs present in non-syndromic HSCR patients may contribute to the phenotype by affecting the regulation or gene-dosage of genes members of those biological pathways involved in the development of the enteric nervous system. Such variations could add up to the phenotypic expression of HSCR. On the other hand, the presence of longer CNVs in syndromic HSCR implies a larger number of disrupted genes, and consequently, a larger number of systems may be affected during developmental stages. Given that the increase in CNV rate was not evident in syndromic HSCR, long CNVs disrupting multiple genes are likely to have a more deleterious effect. One of these genes is likely to implicate in the development of enteric nervous system, particularly the etiology of Hirschsprung disease.

No differences in CNV rate or type were observed when patients were stratified according to the length of the aganglionic segment.

### CNV analysis of syndromic HSCR patients reveals putative candidate genes

Among the 7 large CNVs (>1 Mb) discovered in HSCR patients (Table 2), four were found in unrelated syndromic HSCR patients with mental disabilities (Table 2, in bold). It is interesting to note that three CNVs—a 29 Mb deletion in 11q14.2-q23.2, a 16.68 Mb deletion in 13q21.31-q22.3 and an 11 Mb duplication in 16p11.2-p12.3—coincide with the recently identified candidate regions for intellectual disability (ID) [6], [13]–[15]. In addition, the patient carrying the 16p duplication had also been diagnosed with epilepsy and, incidentally, this 16p11.2 region had also been reported as an epilepsy-implicating loci [16], [17]. While intellectual disability and Hirschsprung disease are frequently associated, it is highly probable that these overlapping regions encompass genes that contribute to the etiology and hence explain the comorbidity of both disorders.

**Table 2:** Large (>1 Mb), rare CNVs identified in HSCR patients.

ID^a^	Additional anomalies	Cytogenetic band	Start	End	Length (Mb)	CN	Candidate genes^b^
NS1	None	8p21.2-p21.3	22,786,027	24,653,171	1.87	3	
**S9**	**Slight mental retardation**	**10q11.22**	**47,030,119**	**48,274,948**	**1.24**	**1**	
**S3**	**Moderate mental retardation; Mild hydrocephalus; Microcephaly; Cardiomyopathy; Congenital hypotonia**	**11q14.2-q23.2**	**85,828,513**	**115,510,680**	**29.68**	**1**	***CNTN5, DYNC2H1, CARD17***
S7	Vertebra anomaly	11q23.2-q25	119,587,696	134,449,982	14.86	1	
**S6**	**Mentally handicapped**	**13q21.31-q22.3**	**61,321,545**	**77,997,415**	**16.68**	**1**	***EDNRB***
NS2	None	15q11.2-q13.1	20,539,701	26,203,954	5.66	3	
**S27** c	**Mental retardation; Epilepsy**	**16p11.2-p12.3**	**16,824,638**	**18,514,090**	**1.69**	**3**	
			**18,728,692**	**28,215,441**	**9.49**	**4**	

a S: Syndromic patient; NS: non-syndromic patient.

b Genes overlapped by recurrent CNVs unique to patients.

c S27 have 2 overlapping duplications within the 16p11-12 locus.

We subsequently examined these regions on other non-syndromic patients, which further revealed 4 smaller deletions encompassed by the 11q14.2-q23.2 CNV. Altogether, several genes were recurrently and uniquely covered by CNVs in patients, including dynein, cytoplasmic 2, heavy chain 1 (*DYNC2H1*) and contactin 5 (*CNTN5*). *DYNC1H1* encodes a cytoplasmic dynein implicated in axonal transport and retrograde trafficking. Mutations in *DYNC2H1* were reported to be associated not only with ID [18] but also with abnormal skeletogenesis which is occasionally found in HSCR [19], [20]. Recent studies in mice also showed that *Dync2h1* mutations disrupted sonic hedgehog (Shh) dependent neural patterning and, most importantly, Shh is essential in gastrointestinal development, plausibly by regulating enteric neural crest cells migration [21]–[24]. Contactin 5, also known as *NB-2*, is a paralog of *DSCAM* and *L1CAM* both previously implicated in HSCR [25]–[28]. All three genes encode neural cell adhesion molecules belonging to the same immunoglobin superfamily. *CNTN5*, together with its paralogs, is involved in the nervous system. It mediates cell surface interactions and the formation of axon connections [29], [30].

Interestingly, the 13q deletion was found to encompass the second major HSCR gene—*EDNRB*. Screening among non-syndromic patients revealed an additional 44 kb deletion disrupting the first exon and intron of *EDNRB*.

Apart from *EDNRB*, none of the other HSCR genes were found to overlap or encompass any copy number changes even if all patients were considered. This result is in line with the negative findings of previous studies on structural variations intersecting selected HSCR-genes [9], [10]. As for other chromosomal regions known to segregate with HSCR (3p21 [31], [32], 19q12, 4q31-q32 [33] and 9q31 [34], [35]), only 2 rare genic CNVs were observed (Table S4). Neither CNV appeared functionally related to the nervous system development nor to the differentiation or migration of neural crest cells. Likewise, no significant overrepresentation of CNVs was found on chromosome 21 [8]. We also investigated chromosomal regions reported altered in HSCR patients as described by the HSCR consortium, including trisomy 21, 10q11 and 13q22 deletions [1]. We found 11 HSCR-specific CNVs intersecting those implicated regions and in particular, 2 CNVs mapped within 10q11 and 3 within 13q22 where *RET* and *EDNRB* reside respectively (Table S5). However, none of the 10q11 deletions encompassed *RET*. Further investigation of the 6 remaining genic CNVs gene(s) is warranted as it may lead to the discovery of new HSCR-susceptibility genes.

### Other genic CNVs

Taken together, a total of 237 non-redundant, rare genic copy number variable regions (CNVRs) were exclusively observed in HSCR patients, corresponding to 246 unique structural variants (see Text S1). To confirm their uniqueness, we compared our HSCR-specific CNVs with the recently published CNV profile on Asian populations [36]. Only two CNVRs were observed in other Asians (Korean or Japanese) and, most importantly, none were observed in the Chinese population. A catalog of these HSCR-specific CNVRs together with the overlapping genes is provided in Table S5. Specially, additional paralogs of *CNTN5*, including *CNTN4*, *SDK1*, *DSCAML1*, *ROBO3* and *ROBO4* were disrupted by HSCR-specific CNVs, highlighting the potential relevance of this immunoglobin family in the development of the disease.

### *NRG3* deletion

**Figure 2:**
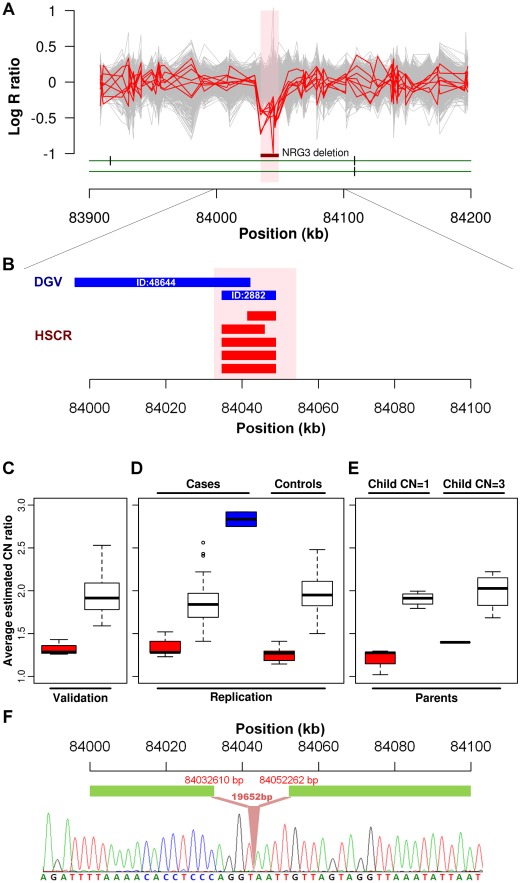
*NRG3* deletions identified in HSCR patients. (A) Intensity signals of 5 HSCR patients (CN = 1; red) with *NRG3* deletions together with other samples of normal copy number (CN = 2; grey). Deleted regions are shown by the dark red bar and are highlighted in pink. (B) Consensus CNV segments of the 5 *NRG3* deletions (red) and the overlapping DGV segments (blue; with DGV ID). (C, D and E) Box plot of *NRG3* copy number estimates by real-time PCR. Samples were grouped according to the called copy number states (CN = 1, red; CN = 2, white; CN = 3, blue); (C) Validation of 5 deletions (CN = 1) and 24 copy-neutral (CN = 2) HSCR patients in the discovery phase; (D) Follow-up analysis on independent case-controls set and (E) Transmission analysis for probands with *NRG3* deletions (child CN = 1) or duplications (child CN = 3). (F) Sequence of the *NRG3* deletion boundary region showing the breakpoint (upstream boundary chr10: 84032610; downstream boundary chr10: 84052262).

We further explored if any particular CNV might be disease causative by comparing the relative frequency in cases to controls. A significant association was found for a CNVR mapping to intron 1 of neuregulin 3 (*NRG3*) located on 10q23.1 (HSCR-CNVR129.1, chr10:84,034,612–84,048,907; hg18; *p* = 1.64×10^−3^). Five hemizygous deletions (3.88%), with estimated length ranging from 8 to 14 kb, were observed in patients (2 syndromic and 3 isolated HSCR patients) while none of the controls had such deletion (Figure 2A). Despite its absence in the controls, it is not a novel CNV. It overlaps with 2 deletions (ID:2882 & 48644) reported for normal population according to the Database of Genomic Variants (DGV, Figure 2B). The former one, with similar boundaries as our cases, has been observed in one HapMap Han Chinese from Beijing (CHB) [37]. A much lower frequency (0.1%) was observed for the latter CNV which extends further upstream [38]. Even though the *NRG3* deletion may not be deleterious, its ten-fold increase in rate for HSCR patients is highly suggestive of pathogenicity. Intriguingly, the deletion encompasses region marked by strong enhancer chromatin signature (H3K4me1) and *DNase*I hypersensitivity, raising the possibility that it might be directly functional (Figure S7) [39].

Neuregulin 3, again, is a paralog of a HSCR-associated gene—neuregulin 1 (*NRG1*; 8p12)—previously discovered in our genome-wide association study utilizing the same intensity data. It encodes a ligand which physically interacts with a transmembrane tyrosine kinase receptor, *ErbB4*. *NRG3*, through activating *ErbB4*, influences neuroblast proliferation, migration and differentiation [40]. Its paralog, *NRG1*, in addition to *ErbB4* also binds to *ErbB2* and *ErbB3*. Meanwhile, a 35 kb *ErbB4* deletion (HSCR-CNVR32.1, chr2:212,872,711–212,889,560) was also observed uniquely in a syndromic HSCR patient.

Due to the biological relevance of *NRG3* in HSCR, we used real-time qPCR to experimentally confirm the deletions. A random set of 46 patients called with normal copy number was chosen for validation. We successfully validated the copy number states of all these samples as shown in Figure 2C. Next, we attempted to replicate this finding on an independent set of 96 cases and 220 controls (Figure 2D). In addition to the 5 deletions found in the discovery phase, nine more deletions (9.38%) were detected in cases while 5 were identified in controls (2.27%) (*p* = 6.92×10^−3^). Seven of those deletions were found in isolated HSCR patients. In addition, real-time qPCR detected 2 novel duplications (2.08%) among the patients included in the replication phase (one patient affected with isolated HSCR and one with HSCR-Meckel diverticulum). All CNVs detected were verified by an additional TaqMan probe. Dissection of sample origin, i.e., from North or South of China, is detailed in Table S6. We found no evidence of association between sample origin and *NRG3* deletion for both cases (*p* = 0.17) and controls (Fisher exact test *p* = 1). To assess if the CNVs are *de novo*, parent-child transmission analysis was performed for those probands (n = 5; 3 with deletion and 2 with duplication) for whom parental DNA was available (Figure 2E). All 3 deletions tested were found to be inherited from normal parents. However, the duplication could not be detected in the parents. Considering deletions alone, we combined association results of both phases, yielding a strong association between HSCR and *NRG3* (*p* = 3.36×10^−5^; *p* = 7.76×10^−6^ when considering all *NRG3* deletions and duplications). Such a 7 fold (6.28% in cases and 0.91% in controls) enrichment of deletions together with *de novo* duplications strongly suggested *NRG3* as a candidate HSCR gene.

Given that most *NRG*3 deletions are inherited, we next attempted to define their nature, i.e. whether they represent a collection of distinct mutations or a low frequency copy number polymorphism instead. In particular, we tried to address two questions, (1) are the deletions on the same haplotype background and if so, (2) do they represent a common ancestral mutation. To achieve this, we performed haplotype analysis on the 206 kb region (chr10:83,990,316–84,195,982) where all 5 typed patients with deletion identified through the GWAS shared at least one allele (i.e. identity-by-state of at least 1). Phasing by BEAGLE revealed a 4-SNP common haplotype harboring the deletion (from rs7085458 to rs7897939; chr10:83,990,316–84,063,139), which suggested a 73 kb identity-by-descent (IBD) segment (Tables S7 and S8). Indeed, this 4-SNP haplotype is the best tagging genetic variant for the deletion, which had a moderate significance of association in our original GWAS data (*p* = 0.005). Identification of the CNV breakpoint (Text S1, Figure S8 and Table S9) revealed a deletion of 19,652 bp in length shared by those 5 patients sharing the haplotype. Close inspection of the boundary sequence, revealed a 4 bp homology between the 5′ and 3′ ends of the deletion. Such microhomologies are observed in 70% of the deletion breakpoints and may reflect the mutational mechanisms leading to the formation of a CNV [41]. These analyses therefore suggest that this deletion involving *NRG3* is a low frequency copy number polymorphism (Figure 2F).

## Discussion

Current data indicates that sporadic HSCR phenotype may result from the interplay and/or accumulation of both common and rare functional DNA variants in genes involved in the enteric nervous system development. These variants may also include structural variations, yet the contribution of CNVs to HSCR had never been investigated at genome-wide level, presumably, because many CNVs are submicroscopic, thus undetectable by conventional karyotyping techniques.

Here we present the first comprehensive survey of copy number variations in HSCR and provide a catalog of rare genic CNVRs possibly implicated in the manifestation of the Hirschsprung disease phenotype. Structural variants identified here are individually rare but collectively common in HSCR patients.

One of the major challenges in CNV discovery is to discriminate between benign and pathological variants. The rarer or longer the CNV, the more likely it is to be pathogenic. Also, the involvement of a gene that lies within a pathway known to contain genes associated with a similar phenotype strengthens the possibility of pathogenicity. Indeed, the CNVs reported here meet the above criteria as we found a plethora of rare CNVs in HSCR patients, in terms of both rate and gene count. In addition, syndromic-HSCR patients were enriched in longer CNV and a number of HSCR-specific CNVs overlapped with paralogs of previously HSCR-implicated genes were observed.

Meanwhile, for rare CNVs, the significant increase in size in syndromic HSCR as well as the overload in number in non-syndromic HSCR suggested a correlation between pathogenesis and genetic heterogeneity at structural level. We attempted to address such correlation across other sub-phenotypes, such as gender and length of aganglionosis. Nonetheless, our study design of random ascertainment limited the sample size of the minor groups and consequently did not permit a detailed investigation of the CNV contribution.

Neuregulin 3 (*NRG3*) encodes a protein similar to its paralog NRG1 and both play important roles in the developing nervous system. As seen with other pathologies, including autism and schizophrenia, several members of a given protein family may associate with the same phenotype, individually or together [7]. Thus far the genes involved in HSCR belong to the two major signaling pathways (RET and EDNRB). The current finding on *NRG3* and *ErbB4* together with our previous study have established the contribution of a new protein family—NRGs—to the disease. Although we have confirmed that both rare and common variants of *NRG1* are associated with HSCR [3], [42], the molecular properties and mechanisms leading to the disease remains unclear. As both *NRG1* and *NRG3* share the same receptor, they may work synergistically or antagonically [43]. Importantly, rare and common variants in NRGs and their receptors have been implicated in schizophrenia [44]–[52]. Furthermore, rare deletions of *NRG1*, *NRG3* and *ErbB4* were described in schizophrenic patients as well [44], [53], [54]. The association of these genes with two different disorders in the nervous system not only emphasizes the relevance of NRGs in the nervous system but also strengthens the validity of the findings. Based on the inheritance we observed, we proposed a two-hit hypothesis for the deletion where the “second hit” could be a mutation or copy number variant in NRG or other inter-related pathways, which explained the incomplete penetrance in parents [55].

One of the intriguing observations from this study is the genetic overlap between Hirschsprung disease and schizophrenia. In addition to the pleiotropic effect of neuregulins and ErbB families, the major HSCR gene, *RET*, was found deleted exclusively in schizophrenia patients in a recent genome-wide CNV analysis [15]. Interestingly, this report, together with Wang *et al.* (2010), also suggested an association between our candidate genes *CNTN5* and schizophrenia [56]. *CNTNAP2*, which was recurrently and exclusively deleted in HSCR cases, was also associated with multiple neurodevelopmental and neuropsychiatric disorders [57]–[59]. It is thus tempting to speculate that pathogenic alterations affecting common pathway(s) may act in the development of both diseases. Such hypothesis is further supported by the frequently observed association of intestinal dysmotility with psychiatric disorders [60]–[62]. Further investigation into the suggestive genetic link is required. By understanding the pleiotropy and the intersecting pathway(s), one can optimize the search for other causal variants underlying HSCR.

Despite none of the known HSCR genes other than *EDNRB* nor HSCR-implicated regions was deleted or duplicated in our analysis, the observation did not elude the presence of structural variations affecting these genes. Rather, it suggested that the rare deletions observed previously, like rare mutations for HSCR patients, might not be a global phenomenon but segregate within individual families.

Similarly, we did not find evidence of global contribution of copy number polymorphisms (CNPs) to HSCR in this study. It could be that indeed these common CNVs are not implicated on the manifestation of the phenotype or that our observation results from the limitations posed by the early genotyping platforms. Our data was generated by *Affymetrix* 500K, well known to suffer from relatively low SNP density and no CNV probes. Regions with CNPs are more likely to violate Hardy-Weinberg equilibrium and could be preferentially excluded from SNP genotyping. Consequently, the power to detect short and/or common CNVs is limited. It should be noted that our stringent quality control to maximize false positive findings in scarify of false negatives also added a further complication to the interpretation of the role of CNPs in HSCR. These limitations applied also to the discovery of the *NRG3* deletions. The higher frequency of copy number changes in the replication samples might only reflect the lower power to detect shorter CNVs with high confidence in the discovery phase.

To conclude, our study provides not only a catalog of rare genic HSCR CNVs but also valuable insights into the contribution of rare CNVs in the phenotypic heterogeneity of HSCR. Our finding illuminates the potential of discovering new HSCR genes and provides grounds for further investigation of the role of NRG family in the disease mechanisms.

## Materials and Methods

### Samples

#### Discovery phase

We started with 173 HSCR Chinese sporadic probands and 340 controls passing SNP-based quality control (QC) as described previously (see Text S1) [3], [35]. All HSCR patients had been screened for the main HSCR genes, namely *RET*, *NRG1*, *EDNRB*, *EDN3* and *GDNF*. Samples were genotyped using *Affymetrix GeneChip 500K* array in which ∼500,000 SNP probes were interrogated separately on two chips (*Nsp* and *Sty*). Further characteristics of the patients can be found in Table S1 and in Garcia-Barcelo *et al.* (2009) and Tang *et al.* (2010) [3], [35]. Only autosomal SNPs were considered in the CNV analysis.

After pre- and post-CNV calling QCs, 129 HSCR cases and 331 controls were left, 29 of whom have additional congenital anomalies in conjunction with Hirschsprung disease. Among these, 8 patients have Down's syndrome and an additional 6 patients with intellectual disability. Details regarding the associated anomalies and known CDS mutations in HSCR genes were listed in Table S2. Out of these 129 patients passing QC, parental DNA was available for 46 probands.

The study was approved by the institutional review board of The University of Hong Kong together with the Hospital Authority (IRB: UW 06-349 T/1374).

#### Replication phase

To replicate our finding on *NRG3* deletion, an independent set of 96 Chinese HSCR cases and 220 controls were subject to the genomic DNA quantification using quantitative real-time PCR. We further determined the inheritance pattern of each *NRG3* CNV discovered (n = 5) for which parental DNA was available.

### CNV calling and quality control (QC)

The overview of CNV calling as well as quality control was summarized in Figure S1 and was detailed in Text S1. Briefly, pre-calling QCs were carried out to remove samples showing relatively low quality in SNP genotyping and samples prone to bias in CNV calling were excluded [63]. Next, CNVs were called by two programs, PennCNV [64] and Birdsuite [65], and were then filtered for abnormal calls. In order to obtain a high-quality CNV dataset, we restricted our analysis to consensus CNV segments consistently called by both programs (Figure S5). Finally, a total of 866 and 1515 CNVs passing quality controls in 129 cases and 331 controls respectively were used for CNV analysis.

### CNV analysis

#### Global CNV burden analysis

CNVs were defined as rare if their frequencies were <1% in the total sample (cases and controls) and were otherwise considered as common. Tests of CNV burden (1-sided) in terms of size, number of CNV segments and number of genes overlapped were performed using permutation by PLINK [66]. Gene annotation was based on UCSC RefSeq (hg18) and NCBI Build 36 was used throughout the study. We defined genic CNVRs as those with more than 1 bp overlapped with any genic region (from −10 kb upstream of the transcription start site to +10 kb downstream).

#### CNV analysis on HSCR gene and HSCR–implicated regions

Four HSCR-implicated regions (3p21 [31], [32], 19q12, 4q31-q32 [33], 9q31 [34], [35]) and 12 HSCR genes (*RET*, *GDNF*, *NRTN*, *SOX10*, *EDNRB*, *EDN3*, *ECE1*, *ZFHX1B*, *PHOX2B*, *TCF4*, *KIAA1279* and *NRG1*) [1] were evaluated for the presence of rare CNVs.

#### Association analysis

Copy number variable regions (CNVRs) were defined as described in Conrad *et al.* (2009) [67] and in Text S1. Two-sided Fisher's exact test was used to test for association between *NRG3* deletions and Hirschsprung disease, both for the discovery and subsequent replication phases. To combine the association results, meta-analysis was performed by pooling the *p*-values while weighted by the sample sizes of each phase.

### Haplotype analysis on the *NRG3* deletion

We phased the genotype calls (1 Mb upstream and downstream) of all 129 HSCR cases and 331 controls using BEAGLE [68], [69]. To increase accuracy (as phasing a small sample set may be somehow inaccurate), we included the unphased genotypes of the 3 carrier parents (from whom NRG3 deletions were inherited) and phased genotypes of 193 Asians (HG00578 was removed due to relatedness) from the 1000 Genomes Project as reference panel [70]. SNPs within the deleted region were recorded as a single bi-allelic marker, with deletion and non-deletion as the two alleles. Association between HSCR and the 4-SNP haplotype encompassing *NRG3* deletion was performed in PLINK.

#### *NRG3* deletion validation and replication

Copy number validation and replication was performed by quantitative real-time PCR (ABI Prism 7900 Sequence Detection System; Applied Biosystems) using TaqMan Copy Number Assay. The assay was carried out in quadruplicates with the TaqMan Copy Number Reference Assay according to the manufacturer's protocol. The reference assay targets a copy-number neural region of RNaseP gene, serving as an internal standard. To achieve high confidence, copy number changes for replication samples were detected and verified by 2 *NRG3* probes (Hs03732951_cn, chr10:84,043,528 & Hs03749105_cn, chr10:84,045,098) which fall within the 4 kb minimal overlapping region of the *NRG3* deletions (chr10: 84,041,355–84,045,997). Relative levels of *NRG3* to reference probes were determined using comparative C_T_ method. In brief, the mean differences in cycle threshold (C_T_) ΔC_T_ between the *NRG3* and the reference probes for all replicates were computed and were subsequently normalized for copy number prediction.

## Supporting Information

Figure S1 Flowchart of CNV discovery and analyses for Hirschsprung disease. Empty boxes indicate number of individuals surviving each step of quality control (QC) while filled boxes designate the procedures for CNV-level discovery and filtering. Hollow arrows denote the CNV exclusion criteria.
(TIF)

Figure S2 PCA plot of normalized intensities in log R ratio (LRR) for the two 500K chips (*Nsp* and *Sty*). The first and second principal components were plotted for (A) by-plate and (B) by-cluster normalized intensities. Batches were represented in gradients of red and blue for HSCR cases and controls respectively.
(TIF)

Figure S3 Box plot of intensity variation parameters for PennCNV. (A) log R ratio variation (LRR SD); (B) BAF drift; (C) median absolute deviation (MAD) and (D) wave factor. Statistical significance between cases and controls was assessed by rank sum test.
(TIF)

Figure S4 Box plot of intensity variation parameters for Birdsuite. (A) copy number (CN) estimate and (B) variation in intensity per chromosome.
(TIF)

Figure S5 Schematic diagram defining the consensus CNV segments. Green and red boxes denote the segments called by Birdseye and PennCNV respectively while consensus CNV was represented by grey shaded box.
(TIF)

Figure S6 Violin plot of CNV rate and gene count for HSCR cases and controls. Shaded regions of the violin plots represent the frequency distribution of CNV rate (upper panel) and gene count (lower panel) for (A,D) all CNVs, (B,E) rare and (C,F) common CNVs. The box in the middle resembles the standard box plot, depicting the lower quartile, median and upper quartile. Samples with more than 30 CNVs (n = 4) are not shown to better illustrate the distribution of the majority.
(TIF)

Figure S7 Functional characteristics at *NRG3* deletion. Enhancer regions implicated by strong signals of chromatin modification H3K4me1 and DNaseI hypersensitivity were shown using corresponding ENCODE tracks in the UCSC genome browser (hg18).
(TIF)

Figure S8 Detection of the *NRG3* deletion breakpoints. Semi-quantitative PCR reactions (Pr1 to Pr8) designed across a 27 kb region spanning the predicted *NRG3* deletion (red vertical stripes on white background) and boundary regions (upstream: green background; downstream: purple background). Pr 4 primer pair was specifically designed within the deletion and used as “deletion-control”. Blue lines: DNA with predicted NRG3 deletion used as template; yellow lines: DNA with no deletion predicted used as template. Primer pair Pr SeqF and PrSeqR was used to amplify the breakpoint once the NRG3 boundaries had been refined by the PCR reactions described. (B): PCR products (1,211 bp) obtained with Pr SeqF and PrSeqR on DNA template from HSCR patients and parents predicted to harbor the *NRG3* deletion and from individuals without deletion (1: Patient HK7, 2: HK7 maternal DNA, 3: Patient HD12, 4: HD12 paternal DNA, 5: Patient HK81, 6: HK81 paternal DNA, 7: Patient HK107; 8: Patient HK122, 9: Individual with no predicted deletion, 10: Individual with no predicted deletion). C: negative control (H_2_O as template). *Denotes amplification with primer pair Pr4 which was used to ensure both DNA quality and PCR efficiency (1,024 bp) on the samples tested. M: 1 kb marker (GeneRuler).
(TIF)

Table S1 Characteristics of the HSCR patients included in the CNV discovery and replication phases.
(DOCX)

Table S2 Genic-CNV and coding sequence (CDS) mutation profile of HSCR syndromic patients.
(DOCX)

Table S3 Relationship between number and length of CNVs
(DOCX)

Table S4 CNVs overlapping HSCR-implicated regions.
(DOCX)

Table S5 List of rare, HSCR-specific genic CNVs.
(XLS)

Table S6 Summary of sample origin for the discovery and replication phase
(DOCX)

Table S7 Haplotypes harbouring *NRG3* deletion for the 5 carriers of the discovery phase.
(DOCX)

Table S8 SNP information of IBS segment shared by 5 HSCR patients with *NRG3* deletions corresponding to Table S7.
(DOCX)

Table S9 Primers and PCR conditions used in the detection of the deletion breakpoint.
(DOCX)

Text S1 Genome-wide copy number analysis uncovers a new HSCR gene: *NRG3*.
(DOCX)
