# Correction: Genome-Wide Copy Number Analysis Uncovers a New HSCR Gene: *NRG3*

**DOI:** 10.1371/annotation/0ffda9e0-ba27-40c1-b766-df58de9083d5

**Published:** 2012-05-23

**Authors:** Clara Sze-Man Tang, Guo Cheng, Man-Ting So, Benjamin Hon-Kei Yip, Xiao-Ping Miao, Emily Hoi-Man Wong, Elly Sau-Wai Ngan, Vincent Chi-Hang Lui, You-Qiang Song, Danny Chan, Kenneth Cheung, Zhen-Wei Yuan, Liu Lei, Patrick Ho-Yu Chung, Xue-Lai Liu, Kenneth Kak-Yuen Wong, Christian R. Marshall, Stephen Scherer, Stacey S. Cherny, Pak-Chung Sham, Paul Kwong-Hang Tam, Maria-Mercè Garcia-Barceló

The eighteenth author's name was incomplete. The complete name is: Stephen W. Scherer. 

