# Supplementary figures and images for: Genome-Wide Copy Number Analysis Uncovers a New HSCR Gene: NRG3
Source: PLoS Genet. 2012 May 10;8(5):e1002687. doi: 10.1371/journal.pgen.1002687 (PMC3349728; doi:10.1371/journal.pgen.1002687)

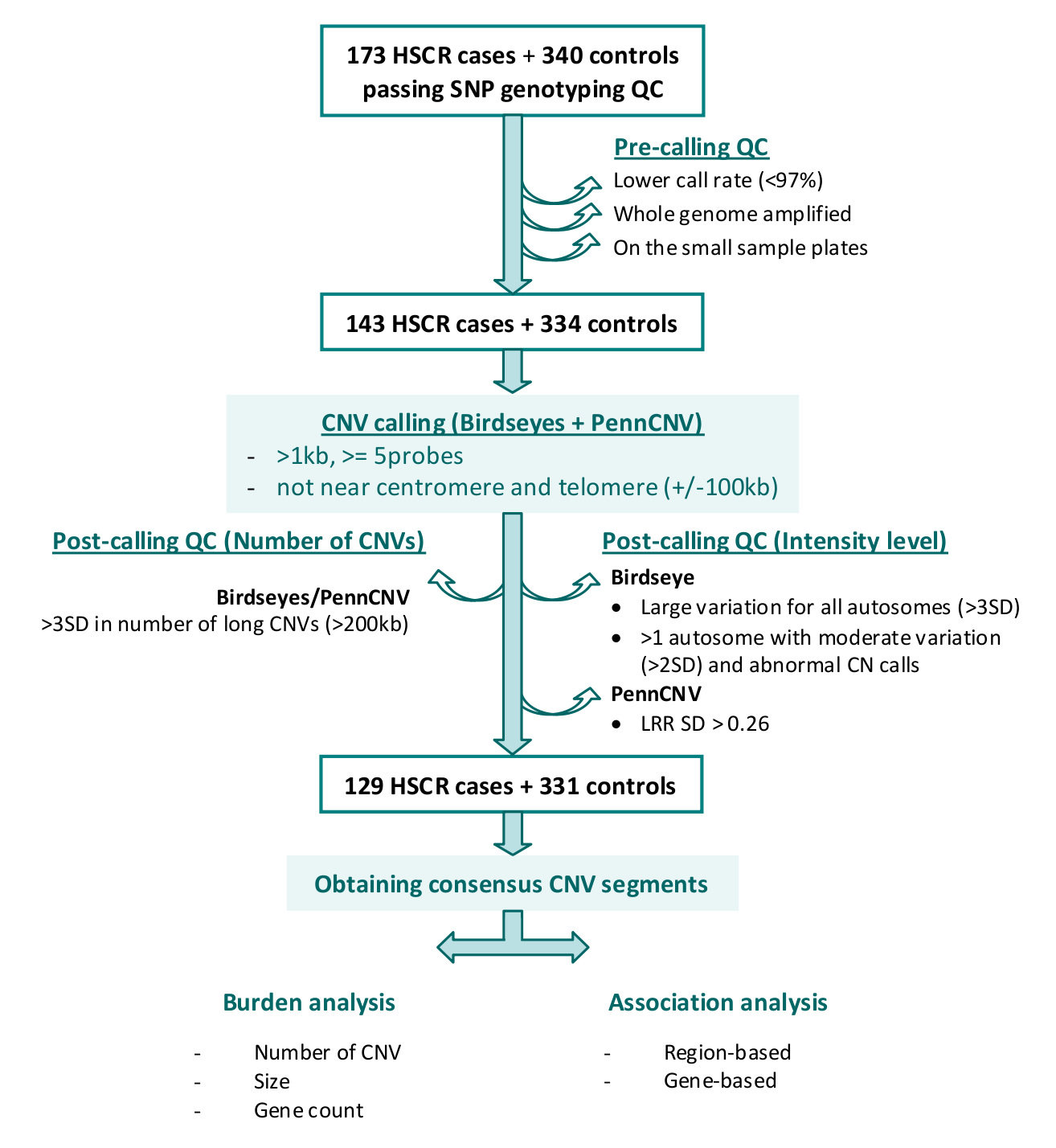

Supplement: Figure S1 — Flowchart of CNV discovery and analyses for Hirschsprung disease. Empty boxes indicate number of individuals surviving each step of quality control (QC) while filled boxes designate the procedures for CNV-level discovery and filtering. Hollow arrows denote the CNV exclusion criteria. (TIF) [file pgen.1002687.s001.tif]

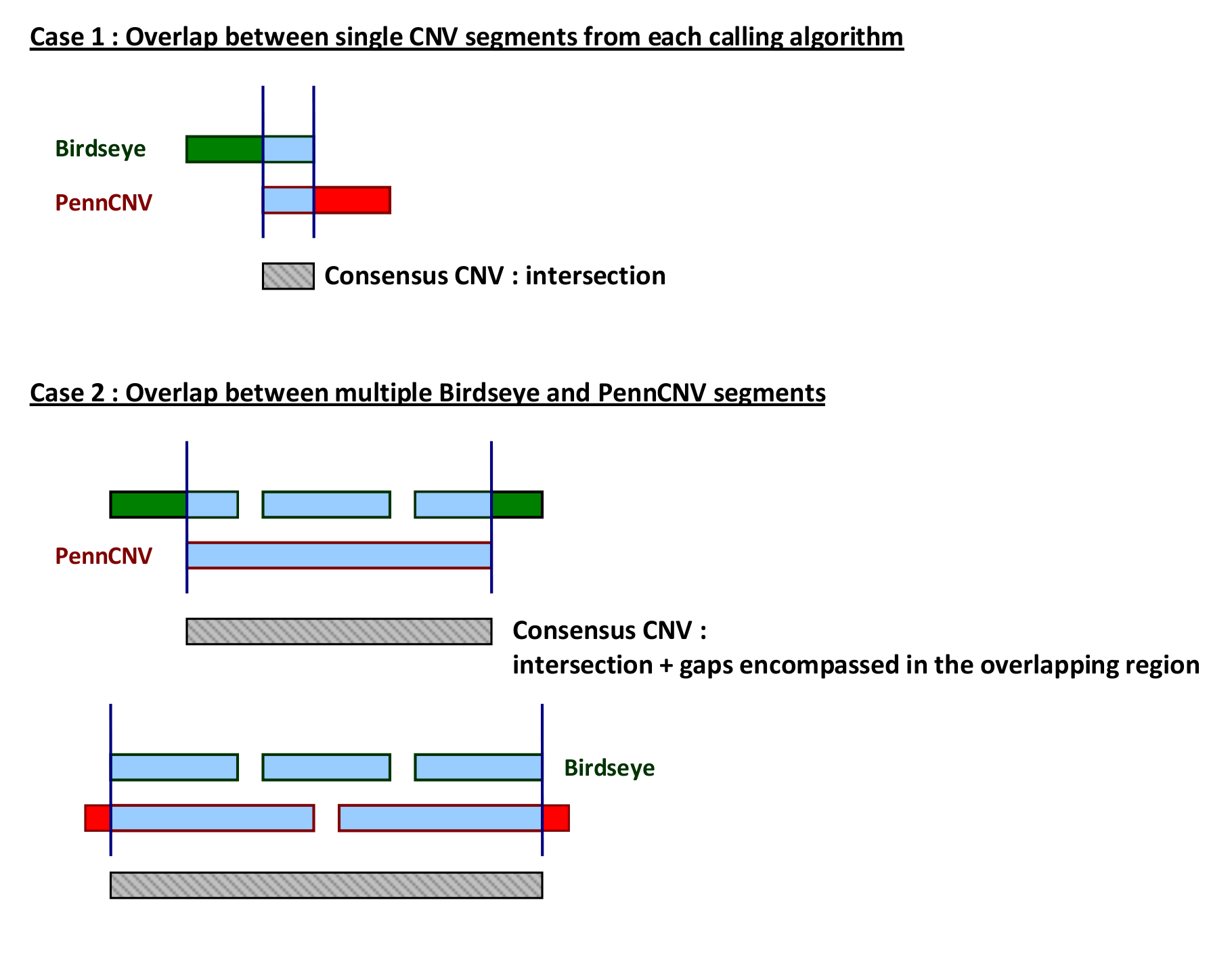

Supplement: Figure S5 — Schematic diagram defining the consensus CNV segments. Green and red boxes denote the segments called by Birdseye and PennCNV respectively while consensus CNV was represented by grey shaded box. (TIF) [file pgen.1002687.s005.tif]

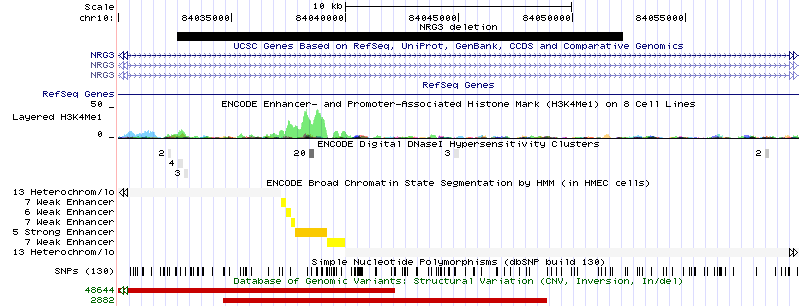

Supplement: Figure S7 — Functional characteristics at NRG3 deletion. Enhancer regions implicated by strong signals of chromatin modification H3K4me1 and DNaseI hypersensitivity were shown using corresponding ENCODE tracks in the UCSC genome browser (hg18). (TIF) [file pgen.1002687.s007.tif]

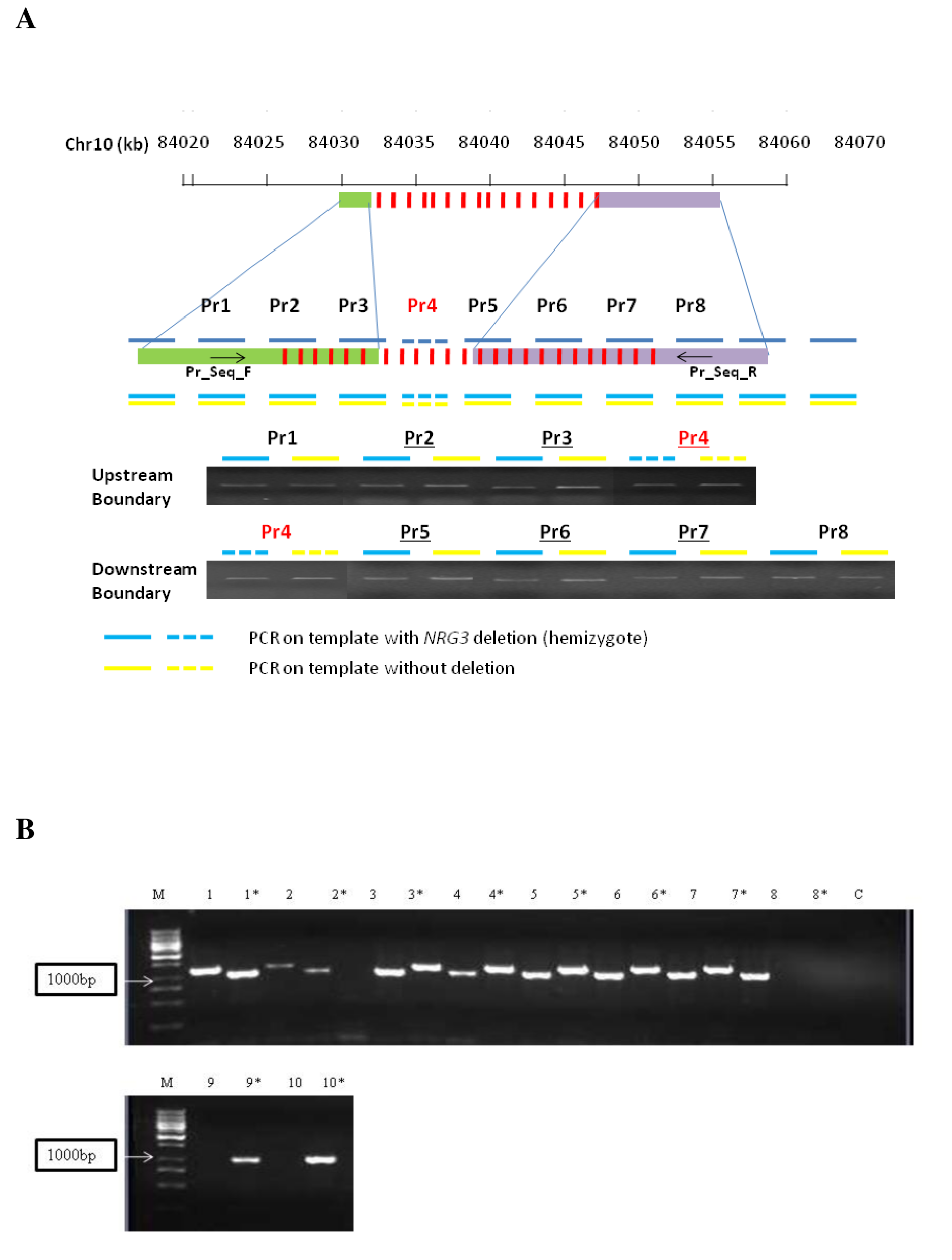

Supplement: Figure S8 — Detection of the NRG3 deletion breakpoints. Semi-quantitative PCR reactions (Pr1 to Pr8) designed across a 27 kb region spanning the predicted NRG3 deletion (red vertical stripes on white background) and boundary regions (upstream: green background; downstream: purple background). Pr 4 primer pair was specifically designed within the deletion and used as “deletion-control”. Blue lines: DNA with predicted NRG3 deletion used as template; yellow lines: DNA with no deletion predicted used as template. Primer pair Pr SeqF and PrSeqR was used to amplify the breakpoint once the NRG3 boundaries had been refined by the PCR reactions described. (B): PCR products (1,211 bp) obtained with Pr SeqF and PrSeqR on DNA template from HSCR patients and parents predicted to harbor the NRG3 deletion and from individuals without deletion (1: Patient HK7, 2: HK7 maternal DNA, 3: Patient HD12, 4: HD12 paternal DNA, 5: Patient HK81, 6: HK81 paternal DNA, 7: Patient HK107; 8: Patient HK122, 9: Individual with no predicted deletion, 10: Individual with no predicted deletion). C: negative control (H2O as template). *Denotes amplification with primer pair Pr4 which was used to ensure both DNA quality and PCR efficiency (1,024 bp) on the samples tested. M: 1 kb marker (GeneRuler). (TIF) [file pgen.1002687.s008.tif]
