# Supplementary material for: Genome-Wide Copy Number Analysis Uncovers a New HSCR Gene: NRG3
Source: PLoS Genet. 2012 May 10;8(5):e1002687. doi: 10.1371/journal.pgen.1002687 (PMC3349728; doi:10.1371/journal.pgen.1002687)
Supplement: Table S1 — Characteristics of the HSCR patients included in the CNV discovery and replication phases. (DOCX) [file pgen.1002687.s009.docx]

| **Supplementary Table 1*:*** Characteristics of the HSCR patients included in the CNV discovery and replication phases | | | | | | | | |
| --- | --- | --- | --- | --- | --- | --- | --- | --- |
| **Discovery^a^**  **(n=129)** | **S-HSCR (n=113)** | | **L-HSCR (n=7)** | | **TCA (n=8)** | | **Undetermined (n=1)** | |
|  | M | F | M | F | M | F | M | F |
|  | 91 {20} (5) | 22 {6} (3) | 2 | 5 | 7 {2} | 1 | 0 | 1 {1} |
| **Replication**  **(n=96)** | **S-HSCR (n=49)** | | **L-HSCR (n=6)** | | **TCA (n=3)** | | **Undetermined (n=38)** | |
|  | M | F | M | F | M | F | M | F |
|  | 44 {8} (4) | 5 {2} | 4 {1} | 2 | 0 | 3 {1} | 36 {1} | 2 |
| ^a^ Number of patients after quality control.  { } additional anomalies; ( ) Down syndrome; M, male; F, female | | | | | | | | |
