# Supplementary material for: Genome-Wide Copy Number Analysis Uncovers a New HSCR Gene: NRG3
Source: PLoS Genet. 2012 May 10;8(5):e1002687. doi: 10.1371/journal.pgen.1002687 (PMC3349728; doi:10.1371/journal.pgen.1002687)
Supplement: Table S2 — Genic-CNV and coding sequence (CDS) mutation profile of HSCR syndromic patients. (DOCX) [file pgen.1002687.s010.docx]

| **Supplementary Table 2:** Genic-CNV and coding sequence (CDS) mutation profile of HSCR syndromic patients | | | |
| --- | --- | --- | --- |
| **ID** | **Additional anomalies** | **CDS mutation(s)^a^** | **HSCR-specific CNV(s)^c^** |
| S1 | Sensorineural hearing loss | None |  |
| S2 (HK107) | Recto-cutaneous fistula  Congenital cystadenomatoid malformation of the lungs | None | **NRG3 deletion (CN=1)**  HSCR-CNVR129.1 |
| S3 | Moderate mental retardation  Mild hydrocephalus  Microcephaly  Cardiomyopathy  Congenital hypotonia | None | **Long deletion (CN=1)**  HSCR-CNVR147.1  **Asian specific deletion (CN=1)**  HSCR-CNVR61.1 |
| S4 | Neurogenic bladder  Down’s syndrome  Bilateral hydronephrosis  Impaired renal function | None | HSCR-CNVR126.1 |
| S5 | Dysmorphic facial features  Microcephaly | None |  |
| S6 | Mental retardation | None | **Long deletion (CN=1)** HSCR-CNVR161.1  HSCR-CNVR132.1 |
| S7 | Vertebra anomalies | None | **Long deletion (CN=1)** HSCR-CNVR149.1  HSCR-CNVR169.1 |
| S8 | Down’s syndrome  Severe bilateral conductive hearing loss | *RET* R114H | HSCR-CNVR87.1 |
| S9 | Slight mental retardation | *RET* IVS11+15(15_36del) | **Long deletion (CN=1)**  HSCR-CNVR124.1 |
| S10 | Renal agenesis | *NRG1* : c.317_319insCGG OR c.316_318insGCG (insert 1 alanine) | HSCR-CNVR86.1 |
| S11 | Down’s syndrome  Flaccid bladder | None |  |
| **Supplementary Table 2 (continued)** | | | |
| **ID** | **Additional anomalies** | **CDS mutation(s)^a^** | **HSCR-specific CNV(s) ^c^** |
| S12 | Chronic liver failure | None | HSCR-CNVR46.1  HSCR-CNVR128.1  HSCR-CNVR136.2 |
| S13 | Down’s syndrome | None |  |
| S14 | Left Inguinal hernia | None |  |
| S15 | Urinary incontinence;  Sacral dimple;  Lipoma | *NRG1* IVS4 -4insA |  |
| S16 | Urinary incontinence | *NRG1* : T41T E53G  *EDNRB* : 5'UT -26 G>A |  |
| S17 | Parathyroid adenoma;  Flaccid bladder  Mental retardation | *GDNF* : R93Q | HSCR-CNVR66.1  HSCR-CNVR203.1 |
| S18 | Moderate sensorineural hearing loss | None |  |
| S19 | Moderate sensorineural hearing loss  Microcephaly | None |  |
| S20 | Down’s syndrome | None |  |
| S21 | Down’s syndrome | None |  |
| S22 | Currarino syndrome | P18*fs*X37in *MNX1^b^* |  |
| S23 (HK81) | Down’s syndrome | None | **NRG3 deletion (CN=1)**  HSCR-CNVR129.1 |
| S24 | Down’s syndrome | None | HSCR-CNVR15.2  **ErbB4 deletion (CN=1)**  HSCR-CNVR32.1  HSCR-CNVR67.1  HSCR-CNVR195.1 |
| S25 | Parathyroid nodules  Prominet left collecting system  Bilateral hydrocele | None |  |
| S26 | Ondine's association | *RET* : 5'UTR -37 G>C | HSCR-CNVR13.1 |
| **Supplementary Table 2 (continued)** | | | |
| **ID** | **Additional anomalies** | **CDS mutation(s)^a^** | **HSCR-specific CNV(s) ^c^** |
| S27 | Mental retardation  Epilepsy |  | **Duplication (CN=3)**  HSCR-CNVR190.1  **Duplication (CN=4)**  HSCR-CNVR191.1 |
| S28 | Mental retardation |  |  |
| S29 | Meckel's diverticulum  Normochromic normocytic anemia |  | HSCR-CNVR74.1  HSCR-CNVR167.1 |
| ^a^:Patients had been screened for variants in the CDS and intron/exon boundaries of *RET,* *EDNRB*, *EDN3*, *GDNF* and *NRG1*.  ^b^:Currarino syndrome susceptibility gene (OMIM 176450).  ^c^:For chromosomal position, please refer to Supplementary Table 4. | | | |
