# Supplementary material for: Genome-Wide Copy Number Analysis Uncovers a New HSCR Gene: NRG3
Source: PLoS Genet. 2012 May 10;8(5):e1002687. doi: 10.1371/journal.pgen.1002687 (PMC3349728; doi:10.1371/journal.pgen.1002687)
Supplement: Table S3 — Relationship between number and length of CNVs (DOCX) [file pgen.1002687.s011.docx]

| Supplementary Table 3. Relationship between number and length of CNVs | | |
| --- | --- | --- |
|  | Correlation coefficient | *P*-value |
| Common |  |  |
| CNVs < 1M | 0.03 | 0.58 |
| All | 0.06 | 0.19 |
| Rare |  |  |
| CNVs < 1M | -0.01 | 0.76 |
| All | -0.01 | 0.79 |
| Syndromic HSCR only | 0.17 | 0.24 |
| Non-syndromic HSCR only | -0.07 | 0.51 |
