# Supplementary material for: Genome-Wide Copy Number Analysis Uncovers a New HSCR Gene: NRG3
Source: PLoS Genet. 2012 May 10;8(5):e1002687. doi: 10.1371/journal.pgen.1002687 (PMC3349728; doi:10.1371/journal.pgen.1002687)
Supplement: Table S4 — CNVs overlapping HSCR-implicated regions. (DOCX) [file pgen.1002687.s012.docx]

| **Supplementary Table 4:** CNVs overlapping HSCR-implicated regions**.** | | | | | | |
| --- | --- | --- | --- | --- | --- | --- |
| **Region** | **Start** | **End** | **CN** | **Length (kb)** | **DGV** | **Nearby gene(s)** |
| 3p21 | 52,466,487 | 52,481,943 | 3 | 15.46 | N | *TNNC1*: troponin C type 1 (slow) (+3.39kb)  *NISCH*: nischarin (0) |
| 4q31-32 | 159,054,799 | 159,076,585 | 1 | 21.79 | N |  |
| 4q31-32 | 162,901,788 | 163,113,553 | 1 | 211.77 | Y | *FSTL5*: follistatin-like 5 (0) |
| 4q31-32 | 167,566,946 | 167,579,350 | 1 | 12.40 | N |  |
| 19q12 | 32,761,177 | 33,492,335 | 3 | 731.16 | N |  |
| 19q12 | 34,559,636 | 34,581,858 | 1 | 22.22 | N |  |
| All 3 CNVs (2 genic) were uniquely observed in HSCR patients. Distance from the nearby genes is shown in brackets. | | | | | | |
