# Supplementary material for: Genome-Wide Copy Number Analysis Uncovers a New HSCR Gene: NRG3
Source: PLoS Genet. 2012 May 10;8(5):e1002687. doi: 10.1371/journal.pgen.1002687 (PMC3349728; doi:10.1371/journal.pgen.1002687)
Supplement: Table S6 — Summary of sample origin for the discovery and replication phase (DOCX) [file pgen.1002687.s014.docx]

| **Supplementary table 6.** Summary of sample origin for the discovery and replication phase | | | | | | | | | | |
| --- | --- | --- | --- | --- | --- | --- | --- | --- | --- | --- |
| Discovery phase (GWAS) | ***NRG3 deletion*** | |  |  |  | | ***NRG3 deletion*** | | |  |
| Northern | YES | NO |  |  | Southern | | YES | NO | |  |
| HSCR | 1 | 48 | 49 |  | HSCR | | 4 | 76 | | 80 |
| Control | 0 | 0 | 0 |  | Control | | 0 | 331 | | 331 |
|  | 1 | 48 | 49 |  |  | | 4 | 407 | | 411 |
|  |  |  |  |  |  | |  |  | |  |
| Replication stage (TaqMan) | ***NRG3 deletion*** | |  |  |  | ***NRG3 deletion*** | | |  | |
| Northern | YES | NO |  |  | Southern | | YES | NO | |  |
| HSCR | 7 | 45 | 52 |  | HSCR | | 2 | 42 | | 44 |
| Control | 3 | 112 | 115 |  | Control | | 2 | 103 | | 105 |
|  | 10 | 157 | 167 |  |  | | 4 | 145 | | 149 |
