# Supplementary material for: Genome-Wide Copy Number Analysis Uncovers a New HSCR Gene: NRG3
Source: PLoS Genet. 2012 May 10;8(5):e1002687. doi: 10.1371/journal.pgen.1002687 (PMC3349728; doi:10.1371/journal.pgen.1002687)
Supplement: Table S7 — Haplotypes harbouring NRG3 deletion for the 5 carriers of the discovery phase. (DOCX) [file pgen.1002687.s015.docx]

| **Supplementary Table 7.** Haplotypes harbouring *NRG3* deletion for the 5 carriers of the discovery phase | | | | | | | | | | | | | | | | | | | | | | | | | | | |
| --- | --- | --- | --- | --- | --- | --- | --- | --- | --- | --- | --- | --- | --- | --- | --- | --- | --- | --- | --- | --- | --- | --- | --- | --- | --- | --- | --- |
|  | **SNPs^a^** |  |  |  |  |  |  |  |  |  |  |  |  |  |  |  |  |  |  |  |  |  |  |  |  |  |  |
| **ID^b^** | **1** | **2** | **3** | **4** | **5** | **6** | **7** | **8** | **9** | **10** | **11** | **12** | **13** | **14** | **15** | **16** | **17** | **18** | **19** | **20** | **21** | **22** | **23** | **24** | **25** | **26** | **27** |
| S2 | **C** | **T** | **G** | **D** | **C** | T | T | A | C | C | A | G | G | T | G | C | T | C | C | G | T | C | C | C | T | G | T |
| NS3 | **C** | **T** | **G** | **D** | **C** | C | C | A | C | T | A | G | A | T | A | C | C | G | C | A | A | T | A | C | T | T | C |
| NS4 | **C** | **T** | **G** | **D** | **C** | C | C | A | C | T | A | G | A | T | A | C | C | G | C | A | A | T | A | C | T | T | C |
| S23 | **C** | **T** | **G** | **D** | **C** | C | C | A | C | T | A | G | A | T | A | C | C | G | C | A | A | T | A | C | T | T | C |
| NS5 | **C** | **T** | **G** | **D** | **C** | C | C | A | C | T | A | G | A | T | A | C | C | G | C | A | A | T | A | C | T | T | C |
| ^a^ SNP IDs are listed in Supplementary Table S7. Highlighted 4-SNP haplotypes represent the shared IBD segments.  ^b^ NS4, S23 and NS5 were phased together with unphased parental genotypes | | | | | | | | | | | | | | | | | | | | | | | | | | | |
