# Supplementary material for: Genome-Wide Copy Number Analysis Uncovers a New HSCR Gene: NRG3
Source: PLoS Genet. 2012 May 10;8(5):e1002687. doi: 10.1371/journal.pgen.1002687 (PMC3349728; doi:10.1371/journal.pgen.1002687)
Supplement: Table S8 — SNP information of IBS segment shared by 5 HSCR patients with NRG3 deletions corresponding to Table S7. (DOCX) [file pgen.1002687.s016.docx]

| **Supplementary Table 8.** SNP information of IBS segment shared by 5 HSCR patients with *NRG3* deletions corresponding to Supplementary Table S7 | | |
| --- | --- | --- |
| **ID** | **rs ID** | **Chromosomal position** |
| 1 | **rs7085458** | 83990316 |
| 2 | **rs11193235** | 84010466 |
| 3 | **rs10884461** | 84029956 |
| 4 | **NRG3del** | 84034612-84048907 |
| 5 | **rs7897939** | 84063139 |
| 6 | rs3908834 | 84068197 |
| 7 | rs11193681 | 84068972 |
| 8 | rs12265675 | 84069725 |
| 9 | rs11193797 | 84083975 |
| 10 | rs7900818 | 84084032 |
| 11 | rs7915217 | 84088095 |
| 12 | rs1937962 | 84110227 |
| 13 | rs7071442 | 84120825 |
| 14 | rs7078253 | 84126661 |
| 15 | rs1937959 | 84136852 |
| 16 | rs12266006 | 84139196 |
| 17 | rs7078757 | 84141992 |
| 18 | rs1937983 | 84142802 |
| 19 | rs12253008 | 84143016 |
| 20 | rs7095137 | 84148528 |
| 21 | rs7095393 | 84148650 |
| 22 | rs11194200 | 84154765 |
| 23 | rs7073820 | 84167004 |
| 24 | rs11194270 | 84173551 |
| 25 | rs11194278 | 84175520 |
| 26 | rs11595839 | 84195058 |
| 27 | rs7909484 | 84195982 |
