# Supplementary material for: Genome-Wide Copy Number Analysis Uncovers a New HSCR Gene: NRG3
Source: PLoS Genet. 2012 May 10;8(5):e1002687. doi: 10.1371/journal.pgen.1002687 (PMC3349728; doi:10.1371/journal.pgen.1002687)
Supplement: Table S9 — Primers and PCR conditions used in the detection of the deletion breakpoint. (DOCX) [file pgen.1002687.s017.docx]

| **Supplementary Table 9.** Primers and PCR conditions used in the detection of the deletion breakpoint | | | | |
| --- | --- | --- | --- | --- |
| Primers | Primer Sequence (5' to 3') | Primer Position | Length | Annealing |
| Pr1_F | GAGCCAGAGGTGCTTTCCCT | 84031421 --84031440 | 1069 | 60^。^C |
| Pr1_R | CTCAGGGGACCACCATGTCC | 84032470 --84032489 |  |  |
| Pr2_F | GATCCTGGCCCCGGTGTTTA | 84032662 --84032681 | 1094 | 60^。^C |
| Pr2_R | ATCGCAATGCTCGCAATGCT | 84033736 --84033755 |  |  |
| Pr3_F | AGCACTGATGTCATCCCCATGGG | 84034155 --84034177 | 846 | 60^。^C |
| Pr3_R | ACCCCCTGACCCAGGTGCTC | 84034981 --84035000 |  |  |
| Pr4_F | AAGGTCCCAGGGAGGGTAGG | 84038852 --84038871 | 1029 | 60^。^C |
| Pr4_R | AGCCCTTCCCAAGGATGACG | 84039861 --84039880 |  |  |
| Pr5_F | GAGTTGGCATAGCAGGCTGC | 84048672 --84048691 | 1014 | 60^。^C |
| Pr5_R | ACACTTTAGGCCGAGCCAGT | 84049666 --84049685 |  |  |
| Pr6_F | GTGAATTGGTTCGAGGCATAGCT | 84050261 --84050283 | 910 | 60^。^C |
| Pr6_R | CCTATTGCTGTCGTGCCTGC | 84051151 --84051170 |  |  |
| Pr7_F | CCATTAAAGGTTACCATCTGCAGC | 84051871 --84051894 | 1054 | 60^。^C |
| Pr7_R | CCCAGGCTTTGAGGTGGAAGA | 84052904 --84052924 |  |  |
| Pr8_F | AAGATGAGTATAAATGCGCTGGGT | 84053286 --84053309 | 1039 | 60^。^C |
| Pr8_R | ACCTCACCTATCCTGACCAATACA | 84054301 --84054324 |  |  |
| Pr_Seq_F | CGAGGGAGCAAGATGGGACA | 84032456 --84032475 | 20826/1211 | 60^。^C |
| Pr_Seq_R | ACCCAGCGCATTTATACTCATC | 84053260 --84053281 |  |  |
