# Supplementary material for: Genome-Wide Copy Number Analysis Uncovers a New HSCR Gene: NRG3
Source: PLoS Genet. 2012 May 10;8(5):e1002687. doi: 10.1371/journal.pgen.1002687 (PMC3349728; doi:10.1371/journal.pgen.1002687)
Supplement: Text S1 — Genome-wide copy number analysis uncovers a new HSCR gene: NRG3. (DOCX) [file pgen.1002687.s018.docx]

**Genome-wide copy number analysis uncovers a new HSCR gene: *NRG3***

Clara Sze-Man TANG, Guo CHENG, Man-Ting SO**,** Benjamin Hon-Kei YIP, Xiao-Ping MIAO, Emily Hoi-Man WONG, Elly Sau-Wai NGAN, Vincent Chi-Hang LUI, You-Qiang SONG, Danny CHAN, Kenneth CHEUNG, Zhen-Wei YUAN**,** Liu LEI, Patrick Ho-Yu CHUNG, Xue-Lai LIU, Kenneth Kak-Yuen WONG, Christian R MARSHALL, Steve SCHERER, Stacey S CHERNY, Pak-Chung SHAM, Paul Kwong-Hang TAM, Maria-Mercè GARCIA-BARCELÓ

***Supplementary Information***

**Content**

[1. Samples 4](#_Toc298503948)

[- HSCR cases 4](#_Toc298503949)

[- Controls 4](#_Toc298503950)

[2. Genotyping and SNP-based quality controls 4](#_Toc298503951)

[3. CNV detection and CNV-based quality controls 5](#_Toc298503952)

[- Pre-calling sample quality controls 5](#_Toc298503953)

[- Intensity normalization 5](#_Toc298503954)

[- CNV calling 6](#_Toc298503955)

[- Post-calling sample quality controls 7](#_Toc298503956)

[- Obtaining consensus PennCNV and Birdseye calls 8](#_Toc298503957)

[4. CNV burden test 8](#_Toc298503958)

[5. Defining copy number variable regions (CNVRs) 9](#_Toc298503959)

[6. Detection of the neuregulin 3 deletion breakpoints 10](#_Toc298503960)

[7. References 11](#_Toc298503961)

**List of Supplementary Figures**

**Figure 1.** Flowchart of CNV discovery and analyses for Hirschsprung disease

**Figure 2.** PCA plot of normalized intensities in log R ratio (LRR) for the two 500K chips

**Figure 3.** Box plot of intensity variation parameters for PennCNV

**Figure 4.** Box plot of intensity variation parameters for Birdsuite

**Figure 5.** Schematic diagram defining the consensus CNV segments

**Figure 6.** Violin plot of CNV rate and gene count for HSCR cases and controls

**Figure 7.** Functional characteristics at *NRG3* deletion

**Figure 8.** Detection of the *NRG3* deletion breakpoints

**List of Supplementary Tables**

**Table 1.** Summary of gender and sub-phenotypes for HSCR patients in the discovery and replication phase

**Table 2.** Rare, HSCR-specific genic CNVs and CDS mutation(s) of HSCR genes identified in syndromic patients

**Table 3.**  Relationship between number and length of CNVs

**Table 4.** CNVs overlapping HSCR-implicated regions

**Table 5.** List of rare, HSCR-specific genic CNVs

**Table 6.** Summary of sample origin for the discovery and replication phase

**Table 7.** Haplotype of the 5 patients with *NRG3* deletion in discovery phase

**Table 8.** SNP information of IBS segment shared by the 5 HSCR patients with *NRG3* deletions corresponding to Supplementary Table S7

**Table 9.** List of primers and PCR conditions for localizing *NRG3* deletion breakpoints

1. Samples

- HSCR cases

All Hirschsprung patients in this study are sporadic (simplex) and are randomly ascertained regardless of segment length, gender, associated anomalies or syndromes. Supplementary Table 1 summarizes the characteristics of the patients used for both the discovery and the replication phases of the analysis.

- Controls

For the genome-wide scan, we employed the same set of controls as described in Tang *et al*. (2010) [[1](#_ENREF_1)]. Replication samples were recruited independently. All controls were of Han Chinese origin.

2. Genotyping and SNP-based quality controls

Samples were genotyped by Affymetrix GeneChip 500K and quality controls were described previously in Tang *et al.* (2010) [[1](#_ENREF_1)] and Garcia-Barcelo *et al.* (2009) [[2](#_ENREF_2)]. Briefly, those with call rate <90% on either chip (Nsp or Sty) or with biological relationship were excluded. For population stratification, samples were clustered using multidimensional scaling (MDS) and nearest neighbor analyses of identity-by-state (IBS) distance as implemented in PLINK [[3](#_ENREF_3)]. We filtered out samples showing evidence of admixture. This confirmed that cases and controls were drawn from the same/single population.

3. CNV detection and CNV-based quality controls

Overview of CNV calling as well as quality control was summarized in Supplementary Figure 1**.**

- Pre-calling sample quality controls

We started with the sample set passing SNP genotyping quality control. To ensure high quality, we further excluded samples prone to bias in CNV calling, such as those 1) with relatively low call rate (<97%), 2) subject to whole genome amplification [[4](#_ENREF_4)], 3) present on the small sample plates and/or 4) from HapMap genotyped on cell-line DNAs. After filtering, 143 HSCR cases and 334 controls remained and were subject to copy number variations detection.

- Intensity normalization

Intensity measurements from all autosomal SNP probes were used to identify deletions and duplications based on the two hidden Markov model (HMM) software, Birdseye [[5](#_ENREF_5)] (of Birdsuite v1.5.3 package) and PennCNV [[6](#_ENREF_6)].

Birdseye

Raw intensities from .CEL files were quantile-normalized across samples typed on the same plate of the same chip (Nsp or Sty) using Affymetrix Power Tools (APT) accompanied by Birdsuite, with parameters “quant-norm.target=1000, pm-only, plier.optmethod=1, expr.genotype=true”. We referred the resulting intensities as by-plate normalized for the rest of this supplementary note.

PennCNV

As PennCNV is primarily based on Illumina platform, intensities must first form three canonical genotype clusters and then transformed into raw CN measures—log R ratio (LRR) and B allele frequency (BAF)—prior to CNV calling.

We evaluated if the by-plate normalized intensities from Birdsuite could be used as input for PennCNV directly. Principal component (PCA) analysis was performed on the transformed LRR (Supplementary Figure 2A); however, 3 clusters were observed for Sty chips, indicating possible batch effect in the by-plate normalized intensities (Supplementary Figure 2A, right panel). While Birdseye counterbalances such batch problem by calling CNVs simultaneously on by-plate basis (thus error in CNV calling would be as minimal as that for SNP genotype calls), PennCNV calls CNVs one individual at a time, irrespective of the similarities among plates. Thus, differences in intensities across plates might introduce biases, leading to false discoveries. To minimize such problem, separate intensity normalization was carried out for PennCNV. Instead of by-plate normalization, we underwent by-cluster normalization for all *Nsp* chips as a whole and for each of the 3 Sty clusters observed in PCA plot. For each cluster (3 for *Sty* and 1 for *Nsp* chips), intensities were quantile-normalized and median-polished with parameters “quant-norm.sketch=50000, pm-only, med-polish, expr.genotype=true” as suggested by the author. The normalized intensities in log2 ratio were later transformed into LRR and BAF for CNV detection. We then performed PCA again on the transformed LRR (Supplementary Figure 2B). This time only one single cluster was observed for both chips, indicating the effectiveness of by-cluster normalization.

- CNV calling

Copy number segments were then detected using Birdseye and PennCNV on the by-plate and by-individual basis respectively. To increase the signal-to-noise ratio, only CNVs longer than 1kb and overlapped with at least 5 probes were considered. As noted from Wang et al. (2007) [[6](#_ENREF_6)], the centromeric and telomeric regions are likely to harbour spurious CNV calls; we thereby further removed any CNV segment with >50% overlap with these regions (100kb+/- ).

- Post-calling sample quality controls

Extensive quality controls were performed on the basis of intensity variation and the number of CNVs called, for Birdseye and PennCNV separately. For Birdseye calls, we first filtered out individuals (3 cases and 2 controls) having abnormally large variation in intensities at genome-wide level (>3 standard deviation (SD) from mean). To avoid removal of chromosomes with truly large copy number variations as observed in the Down’s syndrome patients, we refrained from excluding chromosomes only by abnormal copy number estimates (e.g. CN < 1.75 or > 2.25). Instead, we investigated if these samples also shown large variation in intensity level. Three HSCR cases with at least 2 CN-abnormal chromosomes while having large SNP variance (>2SD from the mean) were considered as having poor CNV-calling quality and the whole samples were left out. These eliminated samples typically had large LRR SD (> 0.26) for the corresponding PennCNV calls. Thus, 3 additional samples (3 cases) with LRR SD > 0.26 were excluded.

Finally, we removed 6 outliers (5 cases and 1 control) in terms of the number of segments called by either PennCNV or Birdseye (number of long CNVs (>200kb) more than 3 SD from the average), resulting in 129 HSCR cases and 331 controls for analysis.

- Obtaining consensus PennCNV and Birdseye calls

Close examination of intensity variation parameters for PennCNV showed that 4 factors, 1) LRR SD, 2) BAF drift, 3) median absolute deviation and 4) wave factor (MAD with direction indicating correlation with local GC content) were significantly larger in QC+ HSCR cases than controls (Supplementary figure 3). On the contrary, similar quality parameter in Birdsuite suggested the reverse (Supplementary figure 4). For Birdsuite, HSCR cases have significantly smaller variation in intensity measures (SNP variance) than controls. To minimize false discovery, we only considered CNVs consistently called by these two algorithms, aiming to compensate the imbalanced intensity variation across cases and controls.

All 8 HSCR patients associated with Down’s syndrome were confirmed with chromosome 21 duplicated (CN=3) by both programs. However, Birdseye tended to artificially split the whole chromosomal duplication into multiple shorter CNVs as a result of the HMM while PennCNV calls were mostly contiguous except for the chromosomal ends (data not shown). Defining the consensus segments as the intersection of calls by the 2 programs would synthetically increase the total number of segments. In view of this, we filled in the gaps between calls as illustrated in Supplementary Figure 5. Such gap filling strategy could avoid the artificial fragmentation while maintaining the overall accuracy, as demonstrated by the absence of correlation between number and size of CNVs (Supplementary Table 3). The resultant minimum physical distance between two neighboring consensus CNVs is 35kb for controls and 82kb for HSCR cases. While the average inter-marker distance for Affymetrix 500K is around 6kb and the sensitivity for calling CNVs>30kb is relatively high, we did not intend to merge any neighboring CNVs together. The final set of consensus segments consisted of 866 and 1515 CNVs for HSCR cases and controls respectively.

4. CNV burden test

Global tests of CNV burden in cases against controls were performed by PLINK. To further confirm the absence of batch effect, we regressed the number of CNVs per individual (CNV rate) on sample plates. Only one plate, which included 2 HSCR cases with more than 30 CNVs, was significantly associated with CNV rate. Such apparent relationship was abolished once these 2 samples were excluded (*p*=0.4823). The association between CNV rate and HSCR remained significant after the exclusion (*p*=2.09x10^-3^ for all CNVs; *p*=2.35x10^-4^ for rare CNVs). Similarly, association between gene count and HSCR persisted. Since patients having more CNVs could be a genuine phenomenon, we further looked into the underlying frequency distribution of CNV rates and gene count. As observed from Supplementary Figure 6, the overall distribution of cases was markedly right-skewed towards higher CNV rate and more overlapping genes when compared to controls, even when we ignored samples with large number of CNVs (>30 CNVs). In line with the higher CNV burden for rare CNVs, the skewness was only observed for the rare, but not for the common CNVs (Supplementary Figure 6B, C, E and F). This confirmed that the observed burden was not primarily driven by a few samples with larger number of CNVs.

It is known that intensity variation positively correlates with CNV rate. To further demonstrate the robustness of the results, we performed additional CNV burden test while conditioning on the intensity variation parameters. Taking a more conservative approach, only the 4 PennCNV factors (LRR SD, BAF drift, MAD and wave factor) indicative of lower data quality in HSCR cases were considered. A stepwise regression was performed with the total number of CNVs as the dependent variable. The best predictive model included 3 of the 4 factors, LRR SD, BAF drift and MAD. We then scored each person by summing the product of each factor with its corresponding effect size (beta, the regression coefficient). Samples were ranked by the score and later grouped into quintile. Conditional permutation for CNV burden was carried out and statistical significances were determined by permuting case-control status within each quintile.

5. Defining copy number variable regions (CNVRs)

To combine CNVs corresponding to the same event, CNV calls were grouped hierarchically into copy number variable regions (CNVRs) similar to that described in Conrad *et al.* (2009) [[7](#_ENREF_7)]. First, any CNVs which overlap more than 1bp were clustered into one CNVR. Then for those CNVs within the same CNVR, any pair with more than 50% reciprocal overlap based on CNV size was grouped into the same sub-CNVR.

6. Detection of the neuregulin 3 deletion breakpoints

To identify the breakpoint, a series of semi-quantitative PCR reactions (Pr1-Pr8; see Supplementary Figure 8A) were designed across a 27 kb region spanning the predicted NRG3 deletion and upstream and downstream boundaries. The Pr4 pair (see Supplementary Figure 8A) was specifically designed within the deleted region and used as deletion control. As DNA template, we used DNAs from one individual predicted to have the deletion (as per GWAS CNV analysis) and from one individual without. These PCR series allowed us to approximately infer the deletion boundaries. Based on the observations from those PCR series, we then designed a PCR reaction (Pr_SeqF and Pr_SeqR) that would only yield product in the presence of the hemizygous deletion (as the “non-deleted” counterpart on the homologue chromosome would be too large to be amplified under the PCR conditions set up). As control for DNA quantity/quality for this latest reaction, PCR using primers Pr_4 that would yield a PCR product of similar size was performed. Sequencing of the Pr_SeqF and Pr_SeqR PCR 1,211bp product revealed the exact breakpoints (Figure 2F). The Pr_SeqF and Pr_SeqR reaction with subsequent sequencing was performed on DNA of those individuals predicted to have the deletion by the GWAS CNV analysis (Supplementary Figure 8B). Primers and PCR conditions are listed in Supplementary Table 9.

7. References

1. Tang CS, Sribudiani Y, Miao XP, de Vries AR, Burzynski G, et al. (2010) Fine mapping of the 9q31 Hirschsprung's disease locus. Hum Genet 127: 675-683.

2. Garcia-Barcelo MM, Tang CS, Ngan ES, Lui VC, Chen Y, et al. (2009) Genome-wide association study identifies NRG1 as a susceptibility locus for Hirschsprung's disease. Proc Natl Acad Sci U S A 106: 2694-2699.

3. Purcell S, Neale B, Todd-Brown K, Thomas L, Ferreira MA, et al. (2007) PLINK: a tool set for whole-genome association and population-based linkage analyses. Am J Hum Genet 81: 559-575.

4. Pugh TJ, Delaney AD, Farnoud N, Flibotte S, Griffith M, et al. (2008) Impact of whole genome amplification on analysis of copy number variants. Nucleic Acids Res 36: e80.

5. Korn JM, Kuruvilla FG, McCarroll SA, Wysoker A, Nemesh J, et al. (2008) Integrated genotype calling and association analysis of SNPs, common copy number polymorphisms and rare CNVs. Nat Genet 40: 1253-1260.

6. Wang K, Li M, Hadley D, Liu R, Glessner J, et al. (2007) PennCNV: an integrated hidden Markov model designed for high-resolution copy number variation detection in whole-genome SNP genotyping data. Genome Res 17: 1665-1674.

7. Conrad DF, Pinto D, Redon R, Feuk L, Gokcumen O, et al. (2010) Origins and functional impact of copy number variation in the human genome. Nature 464: 704-712.
